# Supplementary material for: Growth dynamics and amorphous-to-crystalline phase transformation in natural nacre
Source: Nat Commun. 2023 Apr 20;14:2254. doi: 10.1038/s41467-023-37814-0 (PMC10119311; doi:10.1038/s41467-023-37814-0)
Supplement: Supplementary file 1 — Supplementary Information [file 41467_2023_37814_MOESM1_ESM.pdf]

Supplementary information to:

Growth dynamics and amorphous-to-crystalline phase transformation in natural nacre

L.M. Otter[1*], K. Eder[2], M.R. Kilburn[3], L. Yang[2,4], P. O'Reilly[5], D.B. Nowak[5], J.M. Cairney[2], D.E. Jacob[1]

[1]Research School of Earth Sciences, Australian National University, Canberra, ACT 2601, Australia
[2]Australian Centre for Microscopy and Microanalysis, The University of Sydney, Sydney, NSW 2006, Australia
[3]Centre for Microscopy Characterisation and Analysis, University of Western Australia, Perth, WA 6009, Australia
[4]School of Civil & Environmental Engineering, University of Technology Sydney, Ultimo, NSW, 2007, Australia
[5]Molecular Vista Inc., 6840 Via Del Oro, Suite 110, San Jose, CA 95119, USA

*Corresponding author: Laura.Otter@anu.edu.au

Supplementary Table 1: Summary of data obtained from studied samples.

Analytical approach:	# Specimens and experiments:	Comments:
Sr labelling of live mussels	50 shells from 2 groups (see methods)	Total number of samples from aquaculture experiments
BSE imaging	19 shells, > 5 BSE images/specimen	Survey of a subset of shells
NanoSIMS mapping	8 shells, > 4 maps per specimen	
APT reconstructions	1 shell, 8 APT tips from 2 liftouts	Final data collection using specimen with highest growth rates for best resolution (specimen ID: M2S2R)
PiFM maps	1 shell, 3 maps	
Quantitative EDS maps	1 shell, 1 map	

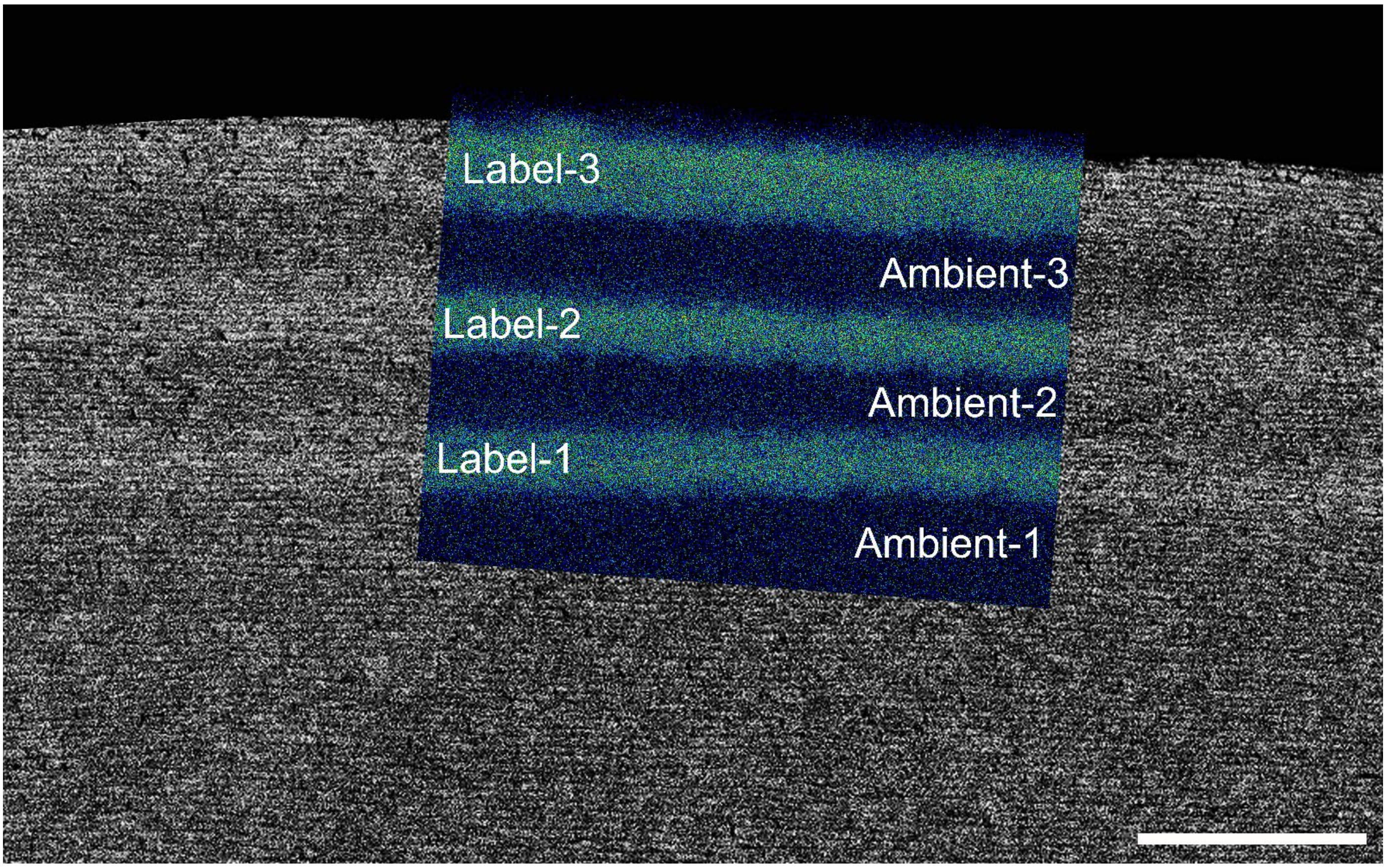

Supplementary Fig. 1. Representative SEM-EDS map (coloured) transecting the three Sr-labels in a *M. galloprovincialis* shell (specimen M2S2R) cross-section overlain on a grey-scale BSE image. Light blue areas (Label-1 to Label-3) formed during Sr pulse-chase labelling intervals in seawater with elevated Sr concentrations, while dark blue map areas (Ambient-1 to Ambient-3) represent shell layers grown in ambient seawater. The three light blue Sr-labelled shell layers correspond to the brighter greyscales of the underlying BSE image. The increased blurriness of the EDS map compared to the BSE image is due to the greater depth at which characteristic X-rays are produced compared to backscattered electrons. Scale bar is 25 µm.

Supplementary Table 2: Element concentrations of the Sr-labelled and unlabelled *M. galloprovincialis* shell portions analysed by quantitative SEM-EDS (data derived from the map shown in Supplementary Fig. 1). The data is presented as averages ± first standard deviations given in µg/g together with the number of spectra (n) for each shell portion as well as the Sr/Ca ratio in mmol/mol. The average Sr concentration, calculated from the three labelled shell portions, is 12,467 µg/g, ($n_{Labelled}$=4,744), while the average Sr concentrations calculated from the three ambient shell portions is 2,033 µg/g ($n_{Ambient}$=6,844).

Region of interest:	Na [µg/g]	S [µg/g]	Ca [µg/g]	Sr [µg/g]	Sr/Ca [mmol/mol]
Ambient-1 (n=2,272)	3,400 ± 100	1,500 ± 100	447,200 ± 100	2,100 ± 100	2.15
Label-1 (n=1,043)	3,700 ± 100	1,000 ± 100	441,300 ± 200	11,900 ± 100	12.3
Ambient-2 (n=1,337)	3,600 ± 100	1,400 ± 100	448,500 ± 100	1,900 ± 100	1.94
Label-2 (n=1,163)	3,700 ± 100	1,300 ± 100	437,500 ± 200	12,500 ± 100	13.1
Ambient-3 (n=3,235)	3,400 ± 100	1,500 ± 100	447,200 ± 100	2,100 ± 100	2.15
Label-3 (n=2,538)	3,200 ± 100	1,400 ± 100	439,600 ± 100	13,000 ± 100	13.53

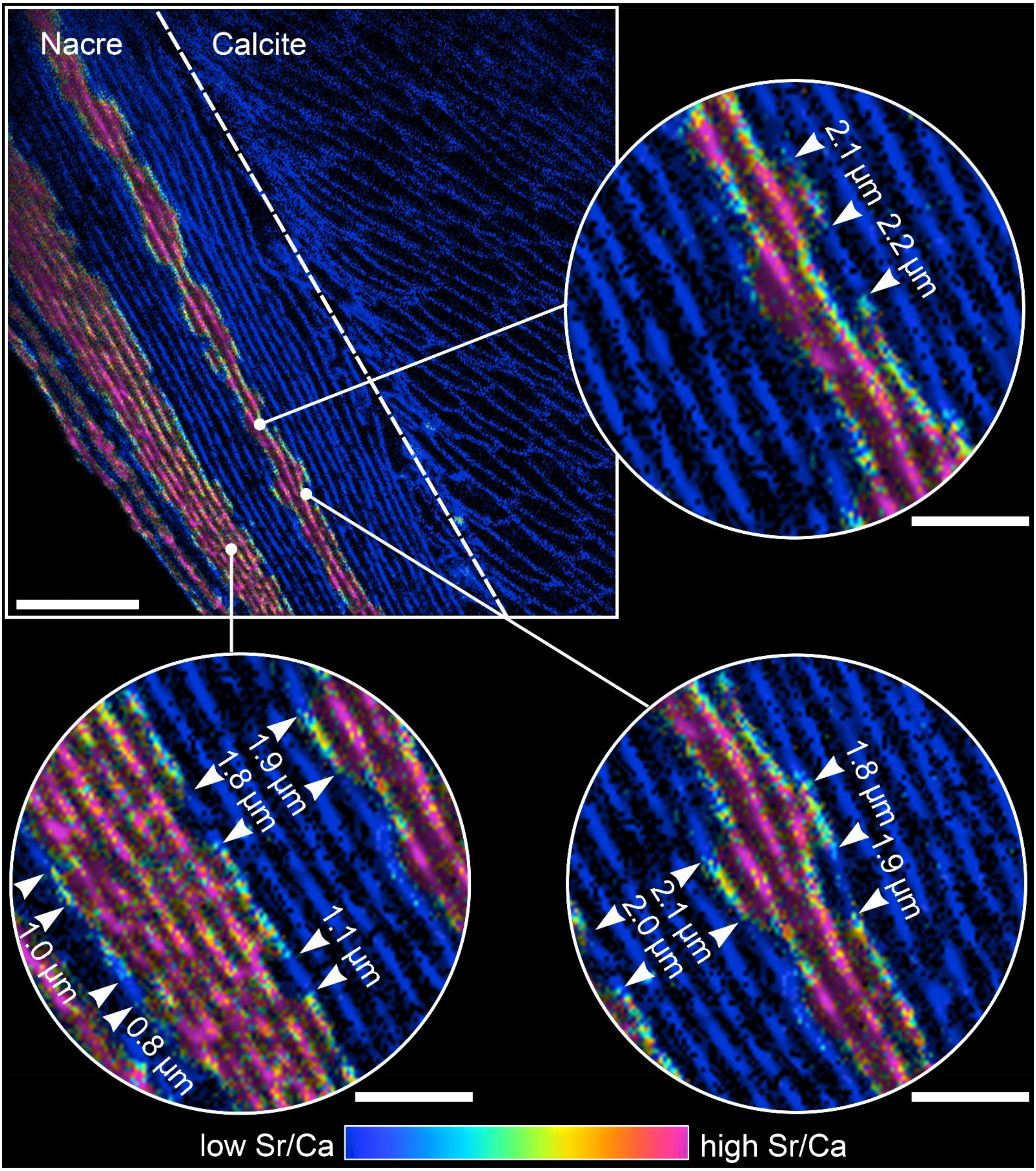

Supplementary Fig. 2. NanoSIMS imaging of nacre growth visualised via Sr pulse chase labelling. Depicted are three additional magnified areas to those shown in Fig. 2 providing snapshots of dynamic nanoscale growth processes (specimen: M1S1L). All magnified regions show a stepped growth pattern of intercalated nacre tablets formed in seawater with normal Sr concentration (blue) and during Sr-labelling (magenta). Magnified regions of interest show sharp (i.e., within 100 nm) near-vertical transitions (white arrowheads) between Sr-labelled and unlabelled portions of nacre lamellae. Some representative neighbouring labelled and unlabelled lamellae portions ranging between 0.8 to 2.2 µm demonstrate that these portions represent changes in Sr/Ca rations within individual tablets as the total length of a nacre tablet is 10-20 µm. Scale bar is 10 µm (large map) and 3 µm (magnified areas).

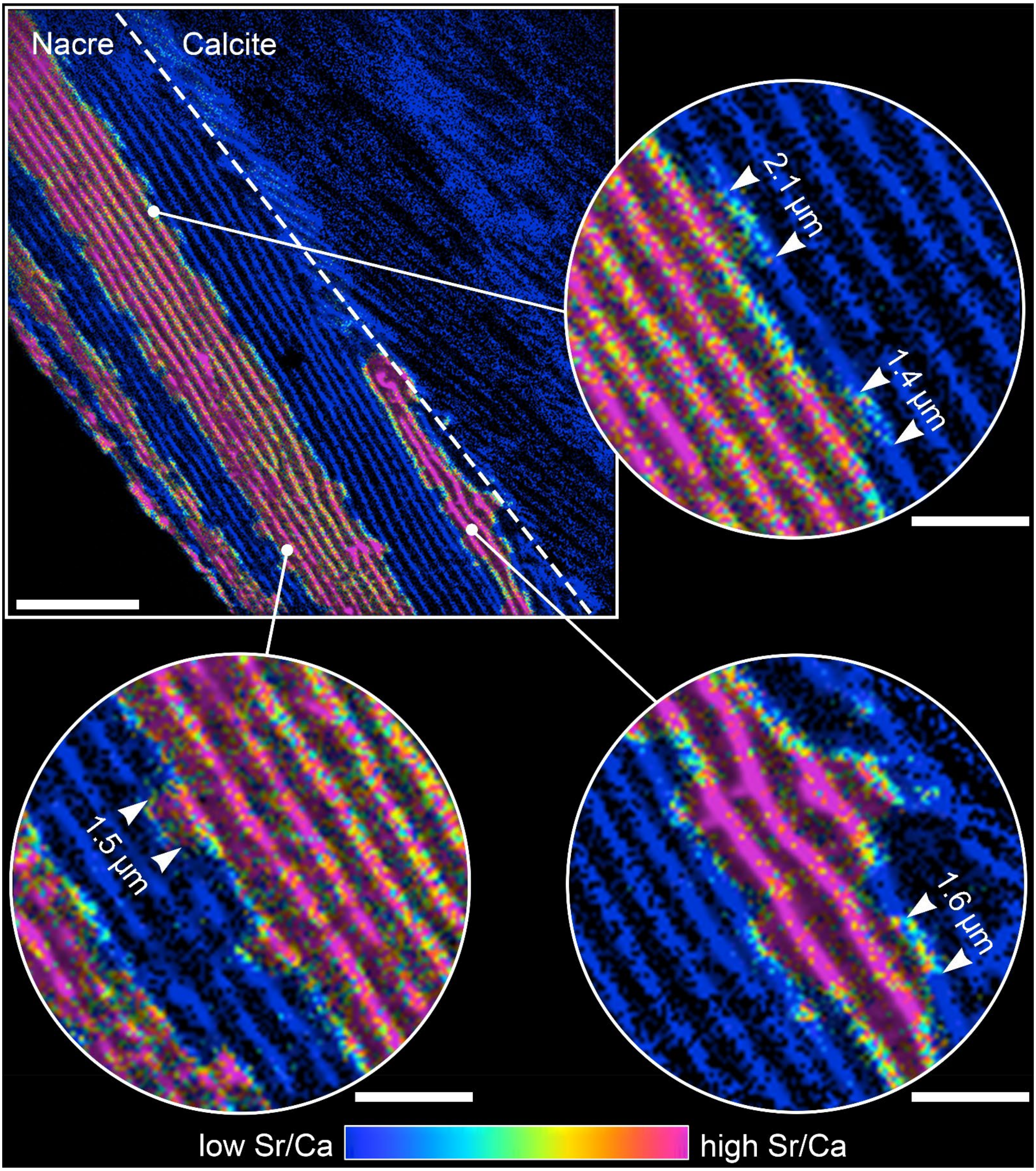

Supplementary Fig. 3. NanoSIMS imaging of nacre growth visualised via Sr pulse chase labelling in an area close to that shown in Supplementary Fig. 2. Depicted are three additional magnified areas providing snapshots of dynamic nanoscale growth processes (specimen: M1S1L). All magnified regions show a stepped growth pattern of intercalated nacre tablets formed in seawater with normal Sr concentration (blue) and during Sr-labelling (magenta). Magnified regions of interest show sharp (i.e., within 100 nm) near-vertical transitions (white arrowheads) between Sr-labelled and unlabelled portions of nacre lamellae. Some representative neighbouring labelled and unlabelled lamellae portions ranging between 1.4 to 2.1 µm demonstrate that these portions represent changes in Sr/Ca rations within individual tablets as the total length of a nacre tablet is 10-20 µm. Scale bar is 10 µm (large map) and 3 µm (magnified areas).

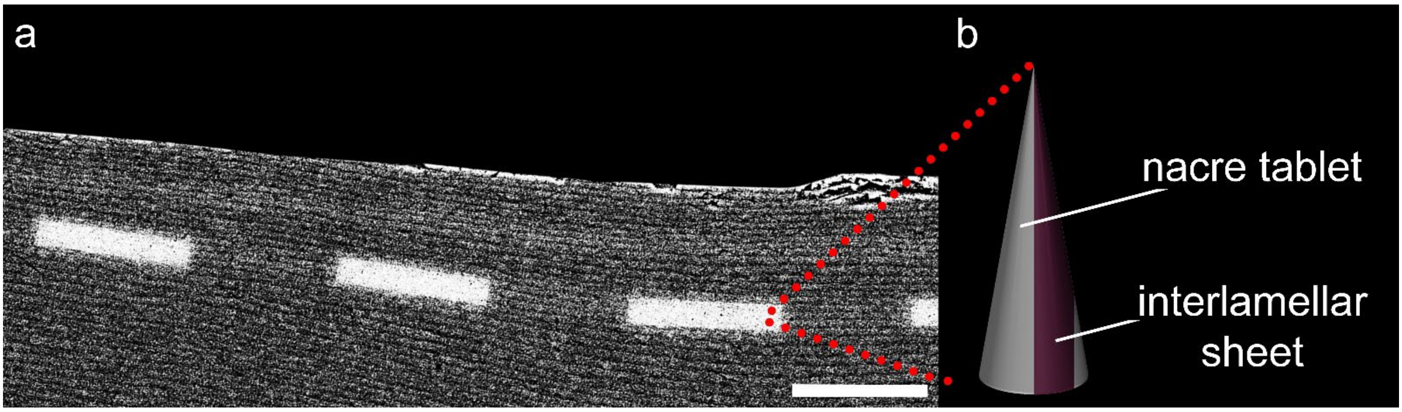

Supplementary Fig. 4: In situ FIB liftout from Sr-labelled nacre for APT analysis. a BSE imaging of the polished nacre cross-section at the umbo used for site-specific FIB-SEM lamellae extractions from within the Sr-labelled nacre (specimen: M2S2R). The selected areas contain the mineral-organic interface at the oganic interlamellar sheet as a point of reference. The protective platinum strips (white stripes in a) were deposited on the Sr-enriched nacre increments prior to wedge milling using a Xe ion beam. b Schematic of a APT tip milled in this study with the organic interlamellar sheet (magenta) close to the tip's apex. Scale bar is 25 µm.

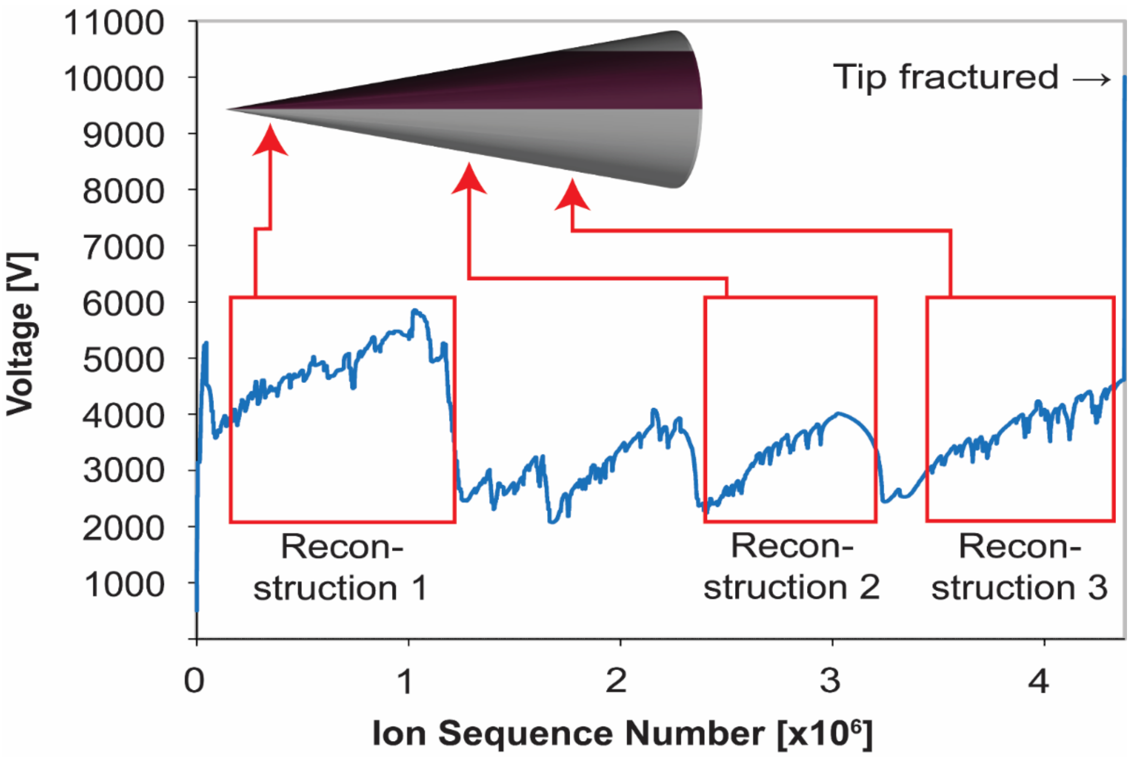

Supplementary Fig. 5. APT Voltage evolution curve for the analysed nacre sample. Bivariate plot showing the voltage evolution during analysis as a function of ion counts for the APT tip. The voltage evolution obtained for nacre (specimen: M2S2R) resembles a saw-tooth pattern due to the repeated encounter of the mineral-organic interfaces, which caused changes in the evaporation field. This is unlike other voltage curves observed for conductive samples where voltage would be observed to ramp up smoothly. We therefore chose to reconstruct the dataset as individual sub-sets where the signal was relatively stable (marked by red boxes and arrows pointing to the approximate location on a schematic APT tip) instead of one large reconstruction to minimise potential artefacts. We selected three areas: area 1 to 3 are shown in Fig. 3, Supplementary Figs. 6 and 8, respectively.

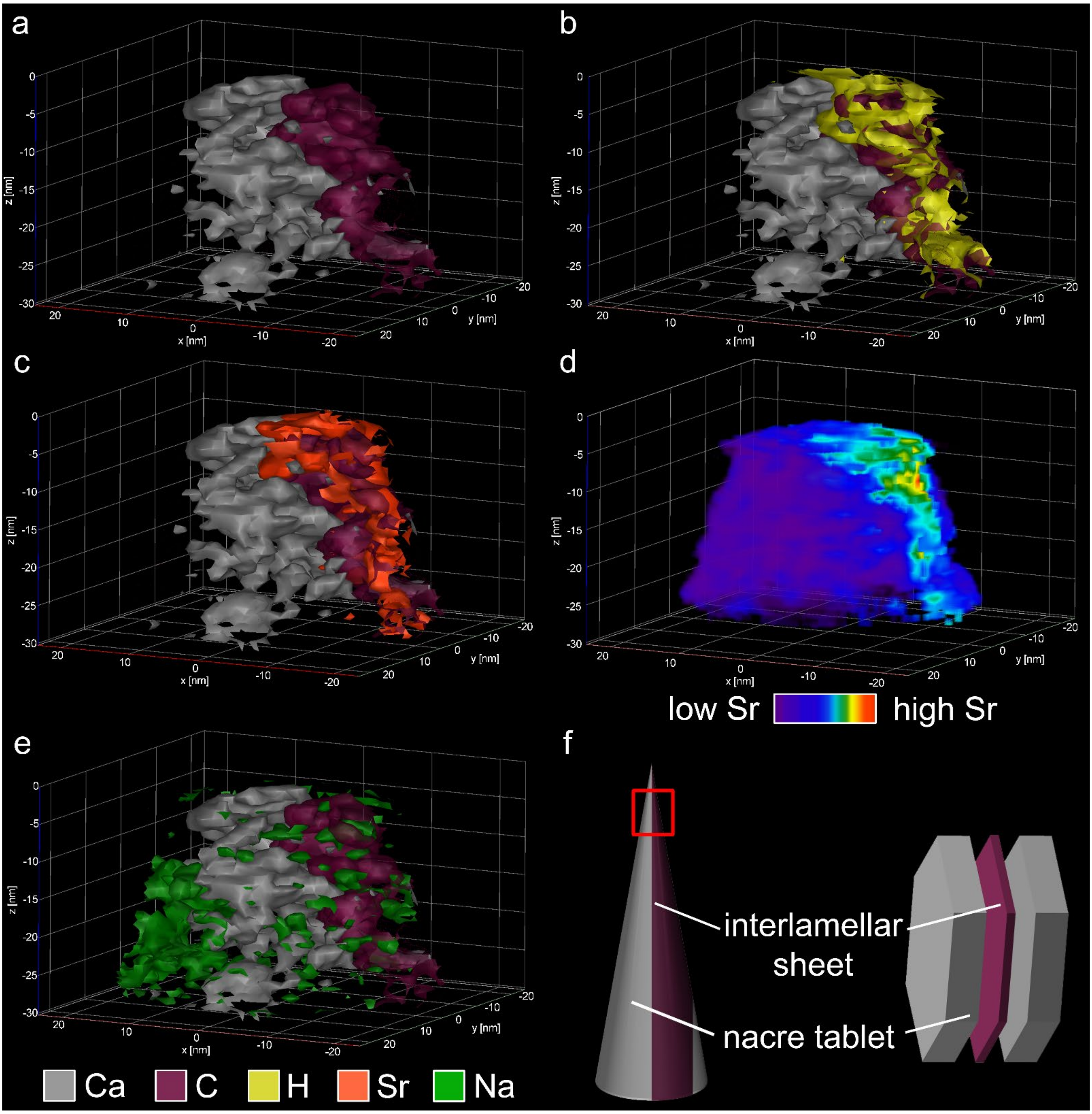

Supplementary Fig. 6. 3D atom probe reconstruction for area 2 in the APT tip showing parts of the nacre tablet and the organic interlamellar sheet in Sr-labelled nacre. a Iso-concentration surface of Ca (50 at%, grey) depicting the outer area of a nacre tablet and C (5.1 at%, magenta) identifies the organic interlamellar sheet. b Iso-concentration surface of hydrogen (10 at%, yellow) co-located with C in the organic interlamellar sheet. c Iso-concentration surface of Sr (20 at%, red) shows enrichment between Ca-rich areas of the nacre tablet. d Volume rendering showing the distribution of Sr across the volume of the reconstruction with enrichments depicted in warmer colours. e Iso-concentration surface of Na (8 at%, green) showing an enrichment inside the mineral phase behind the Sr-enriched area. f Schematic representation of the APT reconstruction (red box) and its relative position within the tip as well as within the nacreous architecture more generally.

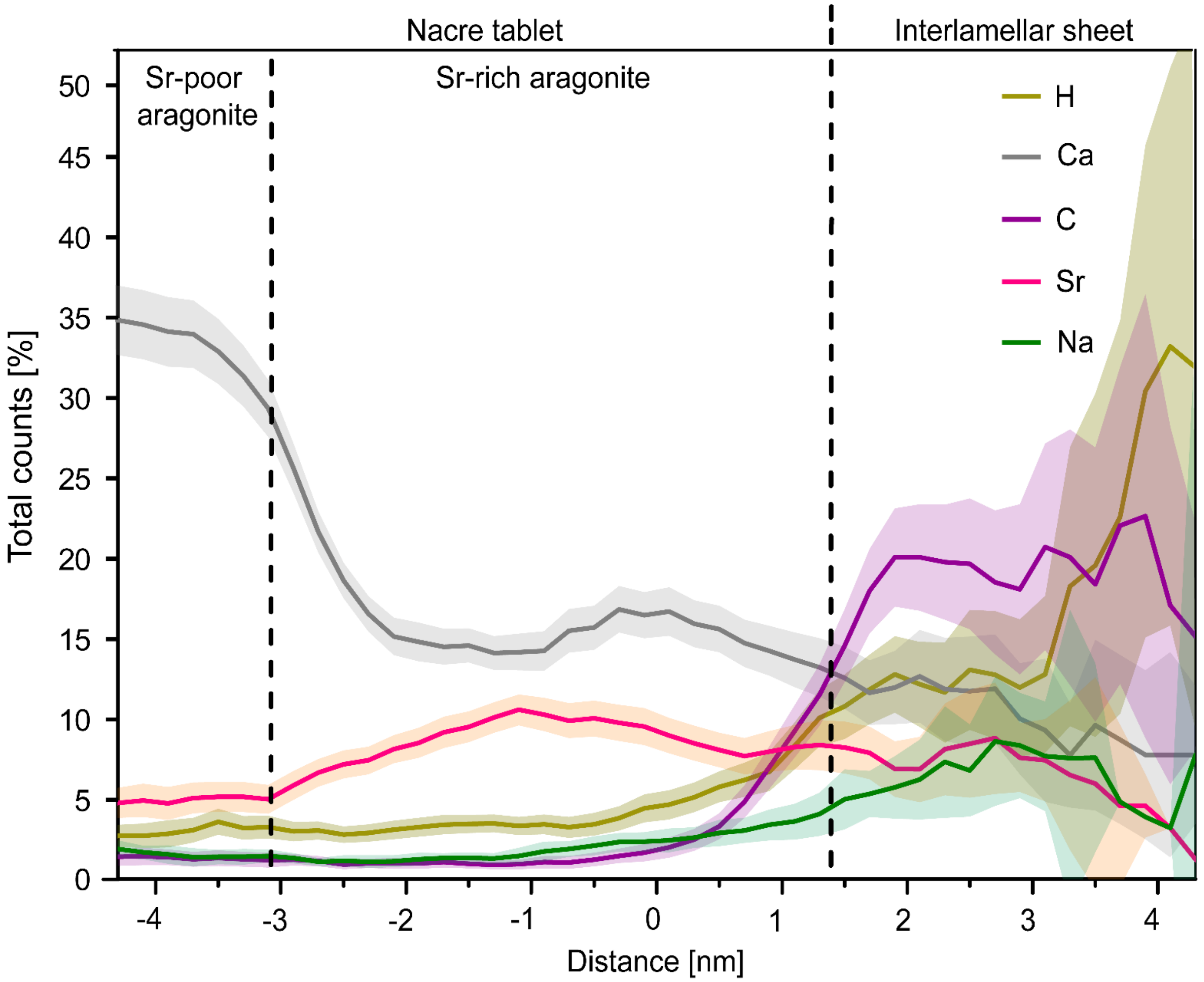

Supplementary Fig. 7. Qualitative compositional line profile across part of the nacre tablet and organic interlamellar sheet (area 2 shown in Supplementary Fig. 6). The profile is divided by black dashed lines into three sections, namely, Sr-poor aragonite, Sr-rich aragonite and interlamellar sheet. The transition from Sr-poor to Sr-rich aragonite is defined by a significant increase in Sr, while the transition from Sr-rich aragonite to the interlamellar sheet is defined as the point of intersection between the Ca (grey) and the C (magenta) signals (see methods). The tablet is characterized by high Ca concentrations indicative of $CaCO_3$, while the Sr-enriched area is defined by high Sr (red) at intermediate Ca and the interlamellar sheet by high C and H (yellow) counts indicative of organics. The Sr-enriched area appears within 4 nm of the mineralized nacre tablet adjacent to the organic sheet. The abundance of Na (green) increases steadily from the tablet into the organic sheet. Shaded envelopes depict first standard deviations.

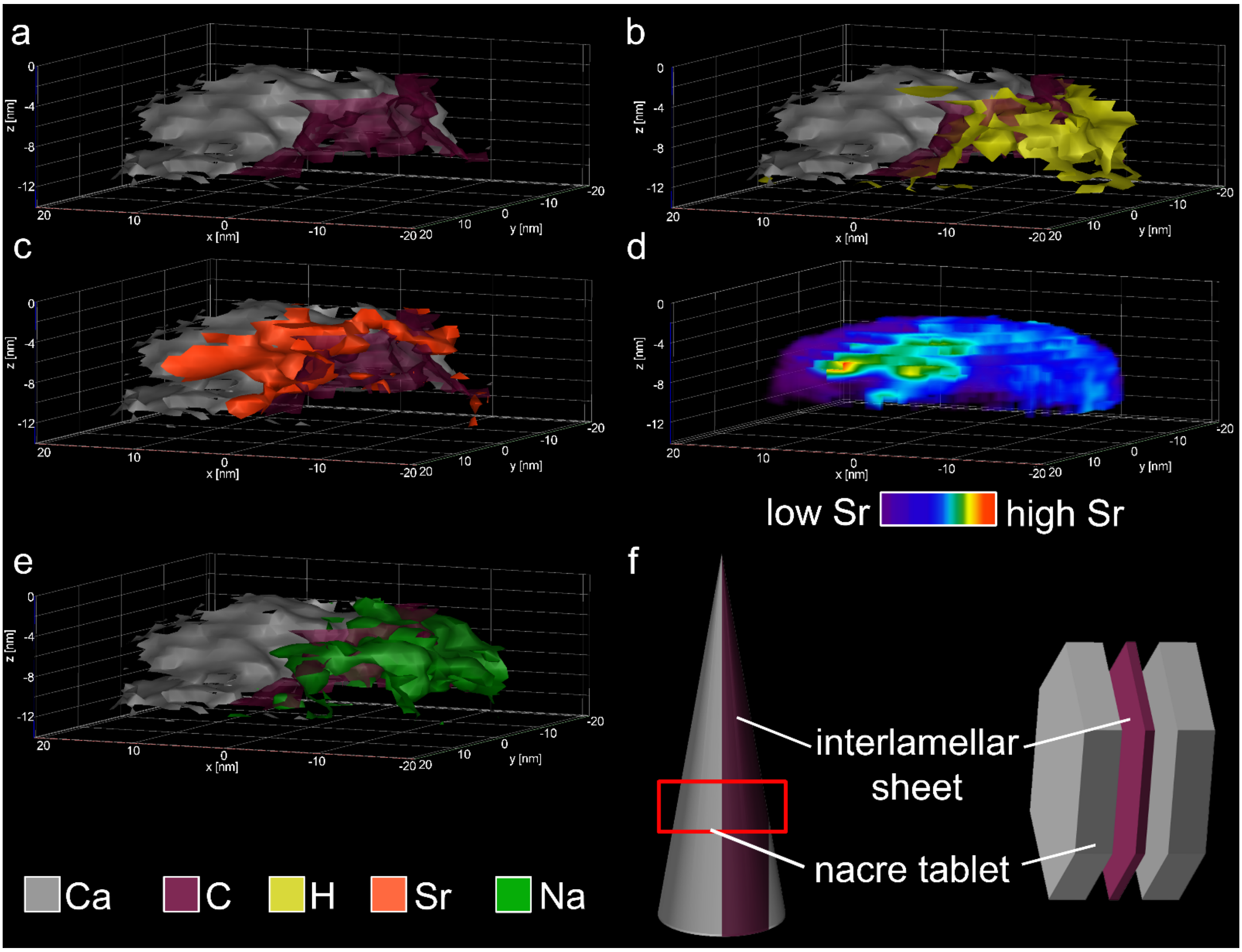

Supplementary Fig. 8. 3D atom probe reconstruction for area 3 in the APT tip showing parts of the nacre tablet and the organic interlamellar sheet in Sr-labelled nacre. a Iso-concentration surface of Ca (50 at%, grey) depicting the outer area of a nacre tablet and C (5.1 at%, magenta) identifies the organic interlamellar sheet. b Iso-concentration surface of hydrogen (10 at%, yellow) co-located with C in the organic interlamellar sheet. c Iso-concentration surface of Sr (20 at%, red) shows enrichment between Ca-rich areas. d Volume rendering showing the distribution of Sr across the volume of the reconstruction with enrichments depicted in warmer colours. e The iso-concentration surface of Na (8 at%, green) showing an enrichment inside the organic phase. f Schematic representation of the APT reconstruction (red box) and its relative position within the tip as well as within the nacreous architecture more generally.

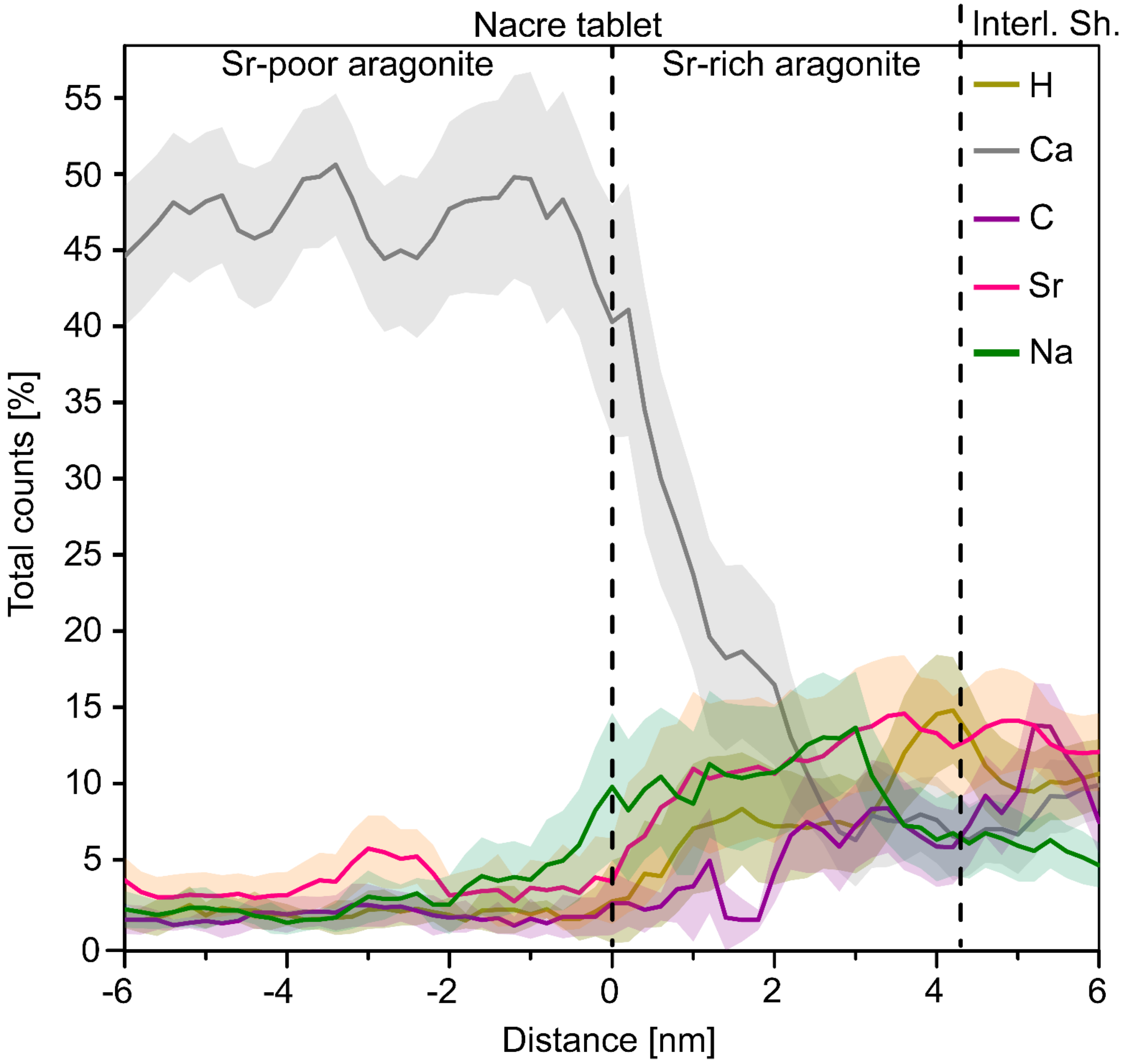

Supplementary Fig. 9. Qualitative compositional line profile across part of the nacre tablet and the mineral-organic interface associated with the interlamellar sheet (area 3 shown in Supplementary Fig. 8). The profile is divided by black dashed lines into three sections, namely, Sr-poor aragonite, Sr-rich aragonite and interlamellar sheet. The transition from Sr-poor to Sr-rich aragonite is defined by a significant increase in Sr, while the transition from Sr-rich aragonite to the interlamellar sheet is defined as the point of intersection between the Ca (grey) and the C (magenta) signals (see methods). The tablet is characterized by high Ca concentrations indicative of $CaCO_3$, while the Sr-enriched area is defined by high Sr (red) at intermediate Ca and the interlamellar sheet by high C and H (yellow) counts indicative of organics. The Sr-enriched area appears within 4 nm of the mineralized nacre tablet adjacent to the organic sheet. The abundance of Na (green) increases steadily from the tablet into the organic sheet. Shaded envelopes depict first standard deviations.

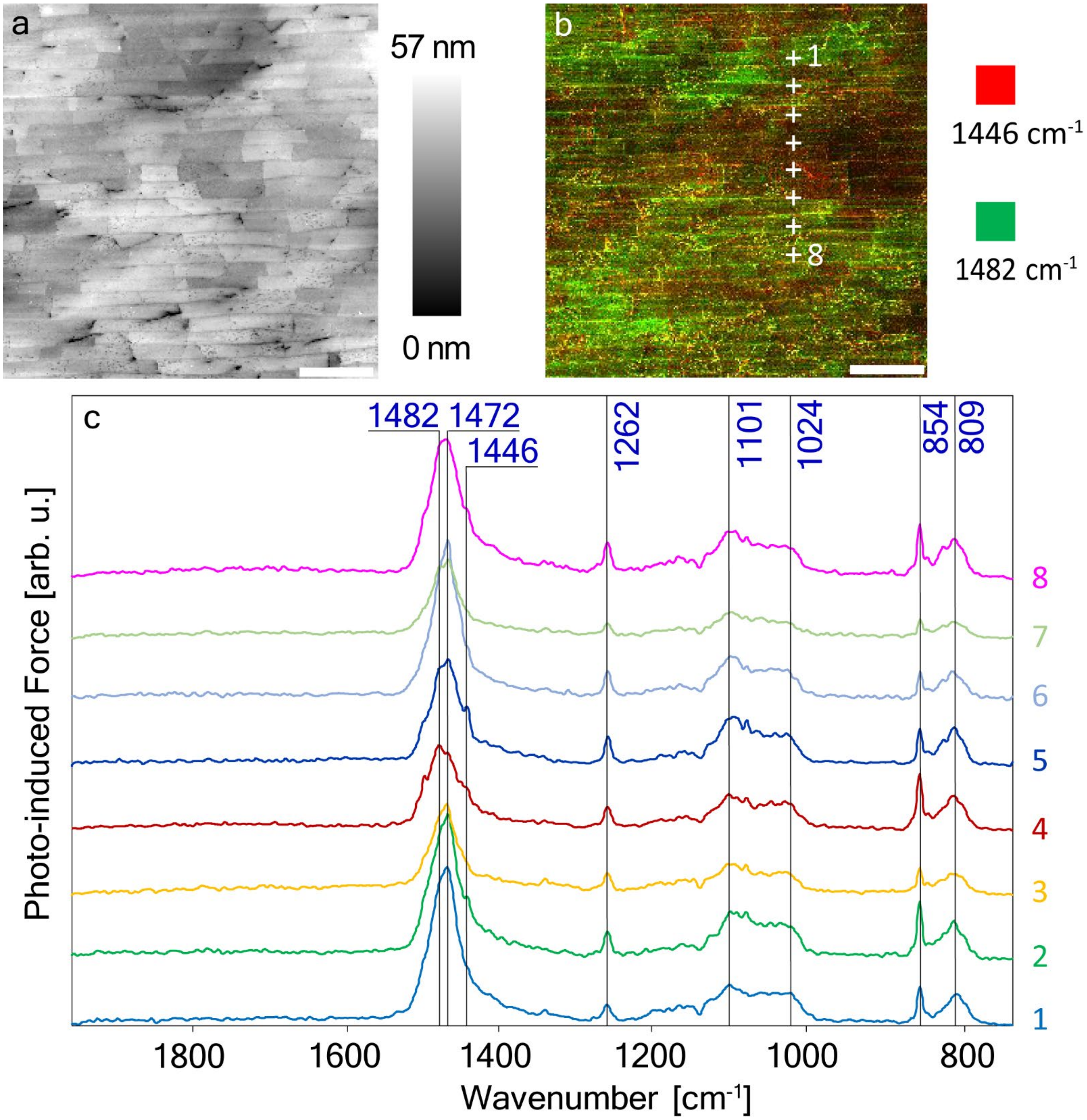

Supplementary Fig. 10. Overview PiFM phase distribution map of Sr labelled nacre (specimen: M2S2R). a Topographic AFM image with lighter and darker colour representing differences in topography (57 nm). b Composite PiFM map obtained via Hyperspectral PiFM IR Imaging (HyPIR) showing aragonite (mapped using the band at 1482 cm^{-1}, green) and a smaller band at 1446 cm^{-1} (red) that appears only within the Sr labelled shell portions and is associated with Sr-rich aragonite. The map was set up to cover the area showing the second Sr-labelled shell layer, which is visible as a red band in the composite map (between the two dashed lines). c PiFM spectra numbered according to their location indicated by white crosses in the composite map. The absorption bands of aragonite (1482 cm^{-1}) and strontianite (1446 cm^{-1}) are clearly visible. The small, sharp bands at 1262 and 854 cm^{-1} are associated with aragonite, while the remaining broad bands are associated with organic phases. Scale bar is 5 µm (a and b).

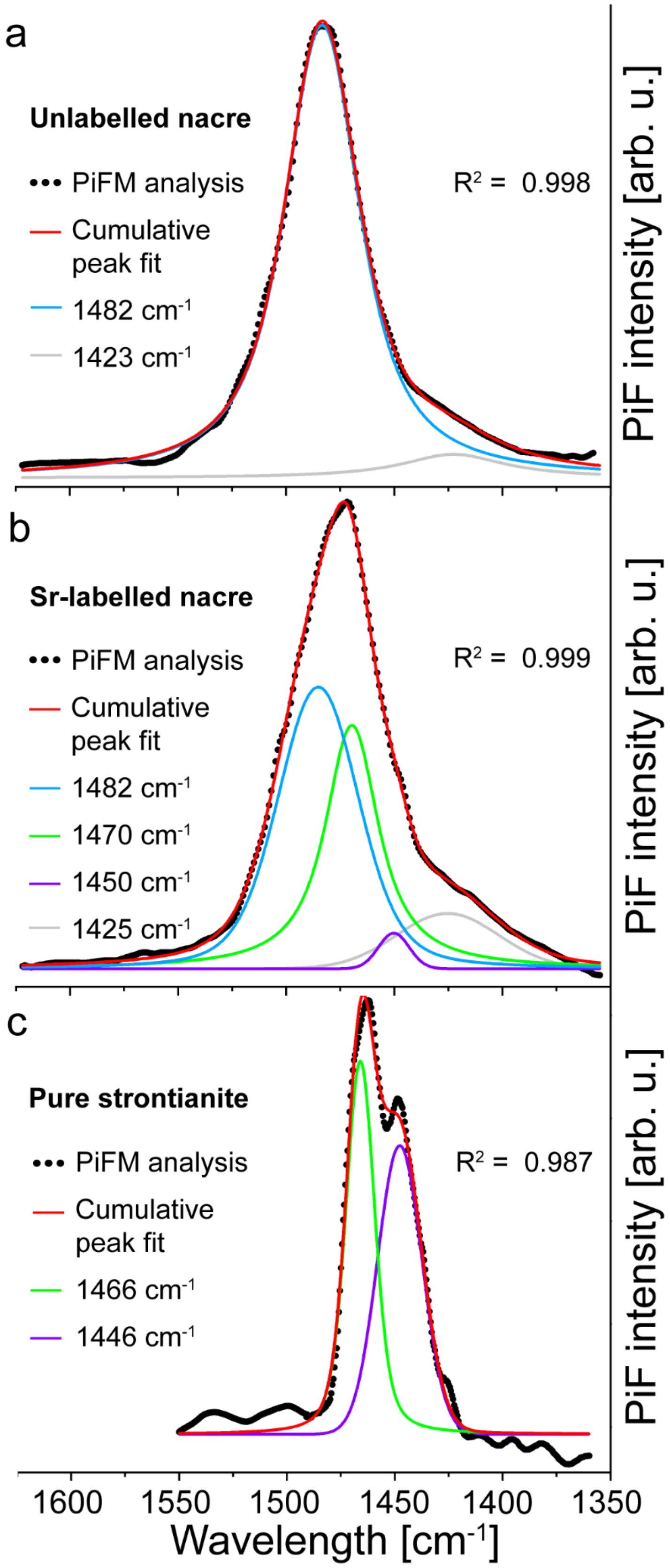

Supplementary Fig. 11. Deconvolved PiFM spectra of nacre and strontianite for comparison in the main absorption band region of carbonate. Averaged PiFM spectra for unlabelled nacre (a) and Sr-labelled nacre (b) and a synthetic strontianite reference material (c, strontianite powder from BDH Laboratory Reagents Dubai, United Arab Emirates). The deconvolution of the pure strontianite spectrum exhibits two peaks: the main peak at 1472 cm^{-1} and a smaller, second one at 1446 cm^{-1} that was observed in the maps shown in Fig. 5, Supplementary Fig. 10 and Supplementary Fig. 11. The deconvolved Sr-labelled nacre (b) shares these two strontianite bands in addition to the aragonite band at 1482 cm^{-1}. The measured spectral envelopes (black dotted lines) were fitted cumulatively (red line) using a Voigt model and the best quality fit (R^2) in OriginPro (version: 2020b, 64-bit).

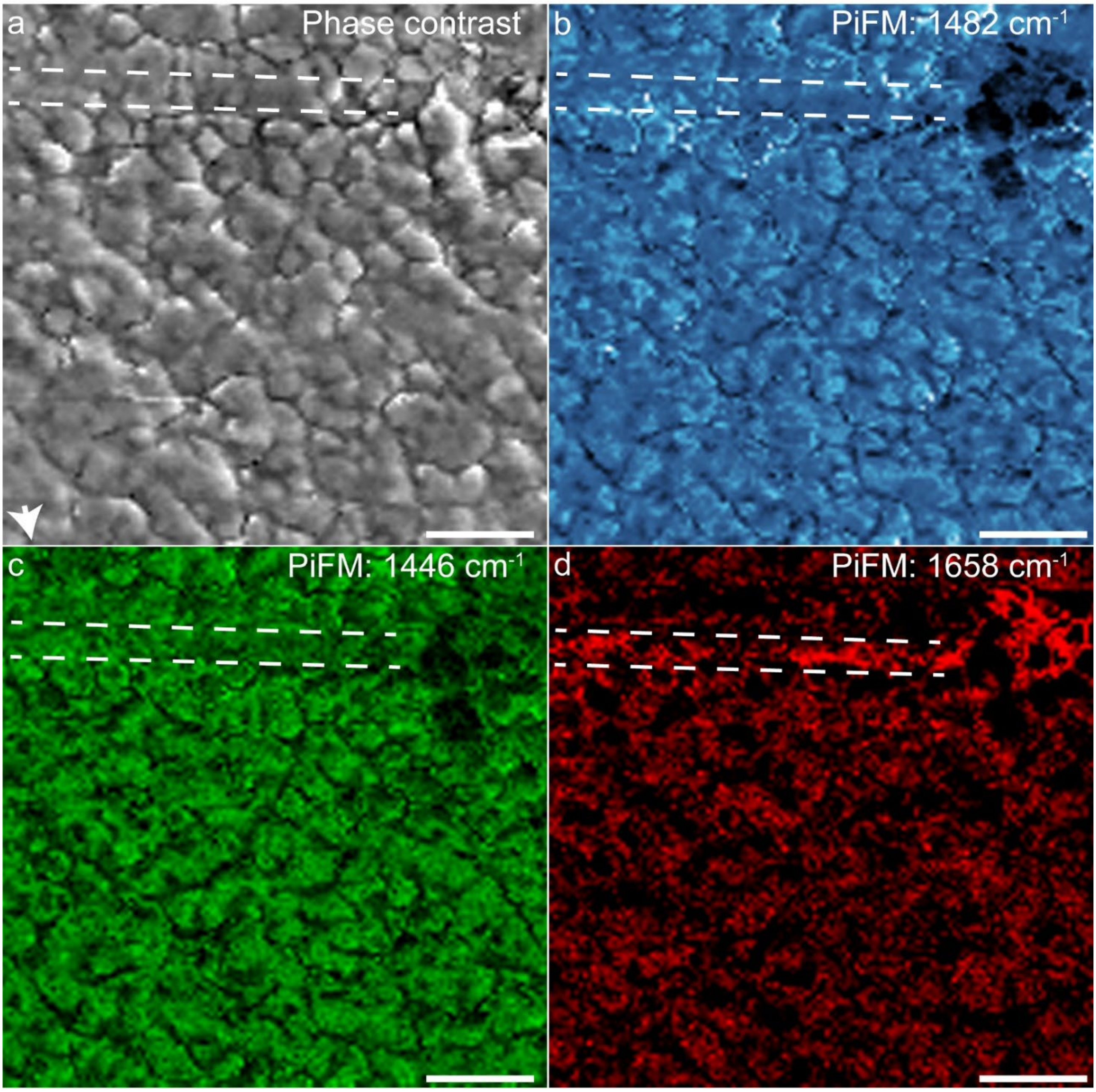

Supplementary Fig. 12. Phase contrast and PiFM phase distribution maps across Sr-labelled nacre from a sample region neighbouring the area shown in Fig. 5. Simultaneously acquired AFM phase contrast and PiFM phase distribution maps sized 0.5 x 0.5 µm obtained from Sr-labelled nacre with the interlamellar organic sheet (within white dashed lines) running nearly horizontally through the mapped region. a AFM phase contrast image with lighter and darker colour highlighting the nanogranular texture, the white arrow points towards the direction of extensional nacre growth (i.e., the inner shell surface) b shows the distribution of the aragonite main band mapped at 1482 cm^{-1} that highlights the nanogranules, c map at 1446 cm^{-1}, which is a small band seen in the spectra of Supplementary Fig. 10, that highlights distinct portions of nanogranules and d phase distribution of the proteinaceous organic phases mapped using the amide I band at 1658 cm^{-1} showing a strong enrichment in the organic sheet and as part of the intracrystalline organic matrix inside the nacre tablet. All four maps were acquired simultaneously in Hyperspectral PiFM IR Imaging (HyPIR) mode and have a pixel resolution of 5 nm. For a PiFM overview map see Supplementary Fig. 10. Scale bars are 100 nm.

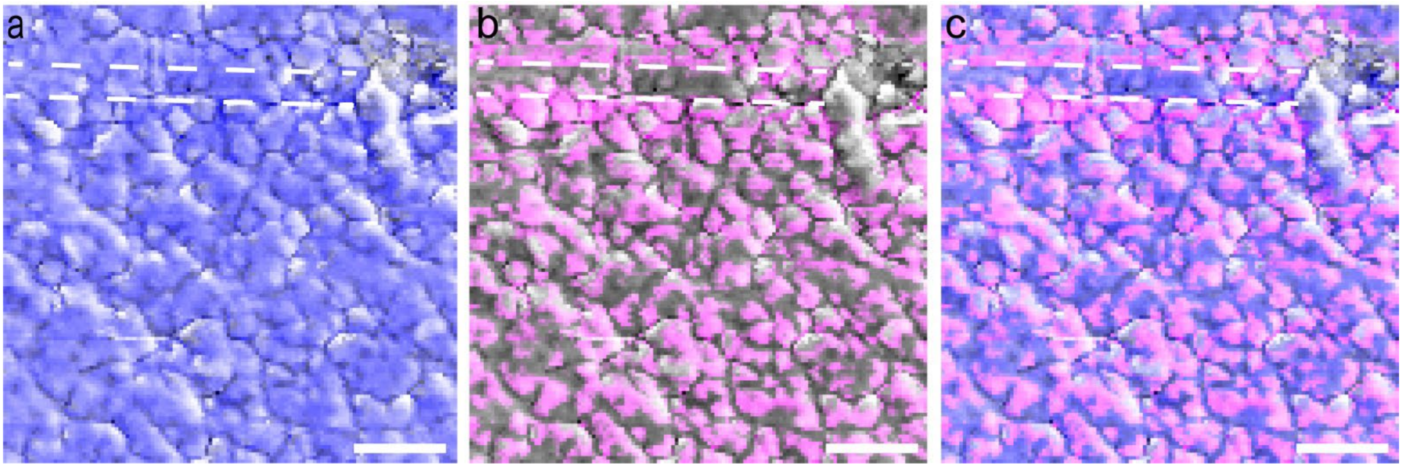

Supplementary Fig. 13. Principal component analysis and multivariate curve resolution (PCA/MCR) maps of Sr-labelled nacre. PCA/MCR component maps obtained from a sample region neighbouring the one shown in Fig. 6 and corresponding to the HyPIR map shown in Supplementary Fig. 12. a component 1 at 1482 cm^{-1} (blue), b component 2 at 1472 cm^{-1} (magenta), and c composite image of component 1 and 2. showing within-granule heterogeneity of separate aragonite and Sr-rich aragonite areas. Sr-rich areas are more commonly observed along the exteriors of individual granules, but rarely form fully enclosed cortices. All three component maps have a pixel resolution of 5 nm and are overlayn on the greyscale phase contrast image showing the space-filling nanogranular texture. Scale bars are 100 nm.
