# Supplementary material for: Growth dynamics and amorphous-to-crystalline phase transformation in natural nacre
Source: Nat Commun. 2023 Apr 20;14:2254. doi: 10.1038/s41467-023-37814-0 (PMC10119311; doi:10.1038/s41467-023-37814-0)
Supplement: Supplementary file 3 — Description of Additional Supplementary Files [file 41467_2023_37814_MOESM3_ESM.pdf]

Description of Additional Supplementary Files

File Name: Supplementary Movie 1

Description: Schematic animation of time-resolved nacre growth based on NanoSIMS maps of Sr-labelled nacre. The labelled nacre sequence shown in the NanoSIMS $^{88}Sr/^{40}Ca$ ratio map (left) is represented in the corresponding simplified schematic animation (right). Strontium labelled nacre is shown in magenta (~12,500 µg/g Sr) and unlabelled nacre in blue (~2,100 µg/g Sr), respectively, while transitions (green and yellow colours seen in the NanoSIMS map) are not shown for simplification. Nacre situated between t_0 (yellow dotted line) and t_1 (green dotted line) formed within 3 days and nacre between t_1 and t_2 (purple dotted line) formed within the following 6 days. This animation demonstrates two sequential growth components for each nacre tablet: extensional nacre growth perpendicular to the interlamellar sheet and space-filling nacre growth parallel to the interlamellar sheets. The animation is in line with previous findings in that extensional nacre growth progresses via mineral bridges across the interlamellar sheets, however, the actual mineral bridges are not shown for simplification. It is suggested that the joining of two tablets leads to the formation of the intertabular sheet (vertical black dashed line). Nacre tablets are shown to extend via space filling nacre growth by maintaining high angle growth fronts as observed directly in our NanoSIMS maps (Fig. 2b, white arrowheads).
