# Supplementary material for: Growth dynamics and amorphous-to-crystalline phase transformation in natural nacre
Source: Nat Commun. 2023 Apr 20;14:2254. doi: 10.1038/s41467-023-37814-0 (PMC10119311; doi:10.1038/s41467-023-37814-0)
Supplement: Supplementary file 5 — Reporting Summary [file 41467_2023_37814_MOESM5_ESM.pdf]

nature portfolio

Corresponding author(s): Laura M. Otter

Last updated by author(s): Mar 13, 2023

Reporting Summary

Nature Portfolio wishes to improve the reproducibility of the work that we publish. This form provides structure for consistency and transparency in reporting. For further information on Nature Portfolio policies, see our Editorial Policies and the Editorial Policy Checklist.

Statistics

For all statistical analyses, confirm that the following items are present in the figure legend, table legend, main text, or Methods section.

n/a	Confirmed	
☐	☒	The exact sample size (*n*) for each experimental group/condition, given as a discrete number and unit of measurement
☐	☒	A statement on whether measurements were taken from distinct samples or whether the same sample was measured repeatedly
☒	☐	The statistical test(s) used AND whether they are one- or two-sided<br>*Only common tests should be described solely by name; describe more complex techniques in the Methods section.*
☒	☐	A description of all covariates tested
☒	☐	A description of any assumptions or corrections, such as tests of normality and adjustment for multiple comparisons
☐	☒	A full description of the statistical parameters including central tendency (e.g. means) or other basic estimates (e.g. regression coefficient) AND variation (e.g. standard deviation) or associated estimates of uncertainty (e.g. confidence intervals)
☒	☐	For null hypothesis testing, the test statistic (e.g. *F*, *t*, *r*) with confidence intervals, effect sizes, degrees of freedom and *P* value noted<br>*Give P values as exact values whenever suitable.*
☒	☐	For Bayesian analysis, information on the choice of priors and Markov chain Monte Carlo settings
☒	☐	For hierarchical and complex designs, identification of the appropriate level for tests and full reporting of outcomes
☒	☐	Estimates of effect sizes (e.g. Cohen's *d*, Pearson's *r*), indicating how they were calculated

Our web collection on statistics for biologists contains articles on many of the points above.

Software and code

Policy information about availability of computer code

Data collection	AZtec 3.1 (SEM-EDS), IVAS 3.6.8 (APT data), NS 50 (NanoSIMS), VistaScan 3 (PiFM)
Data analysis	Open source software: OpenMIMS plugin for ImageJ (NanoSIMS data),<br>Commercial software: AZtec (SEM-EDS), IVAS (APT data), Microsoft Excel (APT and PiFM data), OriginPro (APT and PifM data)

For manuscripts utilizing custom algorithms or software that are central to the research but not yet described in published literature, software must be made available to editors and reviewers. We strongly encourage code deposition in a community repository (e.g. GitHub). See the Nature Portfolio guidelines for submitting code & software for further information.

Data

Policy information about availability of data

All manuscripts must include a data availability statement. This statement should provide the following information, where applicable:

- Accession codes, unique identifiers, or web links for publicly available datasets
- A description of any restrictions on data availability
- For clinical datasets or third party data, please ensure that the statement adheres to our policy

All relevant data supporting the findings of this study are provided in the Supplementary Information file and are available from the corresponding author upon request.

Human research participants

Policy information about studies involving human research participants and Sex and Gender in Research.

Reporting on sex and gender	This is not human research.
Population characteristics	This is not human research.
Recruitment	This is not human research.
Ethics oversight	This is not human research.

Note that full information on the approval of the study protocol must also be provided in the manuscript.

Field-specific reporting

Please select the one below that is the best fit for your research. If you are not sure, read the appropriate sections before making your selection.

☐ Life sciences ☐ Behavioural & social sciences ☒ Ecological, evolutionary & environmental sciences

For a reference copy of the document with all sections, see nature.com/documents/nr-reporting-summary-flat.pdf

Ecological, evolutionary & environmental sciences study design

All studies must disclose on these points even when the disclosure is negative.

Study description	In this work, we use a novel combination of strontium pulse-chase labelling aquaculture experiments and stepwise spatially downscaled correlative analysis to demonstrate for the first time in natural shell samples that nacre forms via localised dissolution and reprecipitation processes within nanogranules. Our study addresses a fundamental question of how amorphous calcium carbonate transforms into the crystalline phase. We use cutting-edge techniques including Atom Probe Tomography, Photo-induced Force Microscopy and Nanoscale Secondary Ion Mass Spectrometry and demonstrate that the transformation occurs via highly localised dissolution and reprecipitation processes rather than by solid state. Hence, our findings elucidate the mechanism of nacre formation and its effects on element distribution at the micron-to-nanoscale for the first time in natural nacre samples.<br>The results open pathways for more realistic trace element and isotope distribution models upon bio-carbonate formation, which are important pre-requisites to improve paleoclimate models. They also explain how the intricate nano-textures in natural biominerals are preserved during a non-classical stepwise crystallization pathway. All data in this study is qualitative except for the SEM-EDS experiment shown in Supplementary Fig 1, which was was ZAF corrected (all parameters listed in the method section).
Research sample	We studied the inner shell layer of the bivalve Mytilus galloprovincialis. The sample was chosen because it contains a nacreous shell ultrastructure. The mussels were sourced from a commercial aquaculture 'Eden Mussels' in Australia. The specimen's sex and exact age are not of interest as these do not impact the shell structure. Juvenile mussels were selected due to their higher shell growth rates compared to adult specimens. This study focuses on the mussel shell and not on any biological aspects related to the mussel soft body, which was discarded.
Sampling strategy	After finalizing the aquaculture experiments, we randomly chose samples that were imaged for determining their growth rates. For micro-and nano-analysis we selected the specimens with the highest growth rates based on electron imaging. We selected specimens for further high-resolution analysis that had the highest growth rates to achieve best spatial resolution with our methods. The exact numbers for each stage of the experiment are provided in Supplementary Table 1.
Data collection	The corresponding author collected the data together with the corresponding analytical specialists, who are all co-authors on this manuscript. The instruments used in this study are SEM-EDS, NanoSIMS, Atom Pobe, and Photo induced Force Microscopy.
Timing and spatial scale	The aquaculture experiments and sample preparation were performed in the period 2016-2017, the NanoSIMS and atom probe experiments were performed in the period 2017-2019, the Pi FM analysis of nacre were performed in 2020 and the Pi FM analysis of pure aragonite and synthetic strontianite were performed in 2022 - we have corrected the typo from the previous version and believe the timing is sufficiently discussed.
Data exclusions	No data were excluded from this study.
Reproducibility	Reproducibility of our results was tested by obtaining multiple dataset for all methods, which are included in the Supplementary info and summarized in Supplementary Table 1.
Randomization	Samples were divided randomely into both groups and later chosen randomly from tanks. Samples for high resolution analysis were chosen based on highest growth rates.
Blinding	Blinding was not relevant for this study as sample selection was based on shell growth rates.

Did the study involve field work? ☐ Yes ☒ No

Reporting for specific materials, systems and methods

We require information from authors about some types of materials, experimental systems and methods used in many studies. Here, indicate whether each material, system or method listed is relevant to your study. If you are not sure if a list item applies to your research, read the appropriate section before selecting a response.

Materials & experimental systems

n/a	Involved in the study
☒	☐ Antibodies
☒	☐ Eukaryotic cell lines
☒	☐ Palaeontology and archaeology
☐	☒ Animals and other organisms
☒	☐ Clinical data
☒	☐ Dual use research of concern

Methods

n/a	Involved in the study
☒	☐ ChIP-seq
☒	☐ Flow cytometry
☒	☐ MRI-based neuroimaging

Animals and other research organisms

Policy information about studies involving animals; ARRIVE guidelines recommended for reporting animal research, and Sex and Gender in Research

Laboratory animals	Bivalves do not fall under ethics approval. We used the bivalve mollusk Mytilus galloprovincialis. Strain and age were not determined as they are not relevant for this study.
Wild animals	This study used bivalves sourced from marine aquaculture and are thus no wild animals.
Reporting on sex	Sex was not determined and not of relevance to the study.
Field-collected samples	The samples were collected from a commercial aquaculture provider and were thus not collected in the wild.
Ethics oversight	Bivalves do not fall under ethics approval

Note that full information on the approval of the study protocol must also be provided in the manuscript.
